# Rhegmatogenous retinal detachment associated with spontaneous suprachoroidal hemorrhage in high myopia patients: two case reports and systematic review

**DOI:** 10.1007/s10792-025-03925-4

**Published:** 2025-12-23

**Authors:** Xiao-Xin Liang, Chi-Xin Du, Long-Bin Yu, Wei-Na Ren, Yan Sheng

**Affiliations:** 1 Department of Ophthalmology, The Fourth Affiliated Hospital of School of Medicine, Zhejiang University, Yiwu, Zhejiang 322000 China; 2 Department of Ophthalmology, The First Affiliated Hospital of School of Medicine, Zhejiang University, Hangzhou, Zhejiang 310003 China; 3 Department of Ophthalmology, Fuyang First People’s Hospital, Hangzhou, Zhejiang 310003 China; 4 Department of Ophthalmology, The Eye Hospital of Wenzhou Medical University (Ningbo Branch), The Affiliated People’s Hospital of Ningbo University, Ningbo, 315040 China

**Keywords:** Spontaneous suprachoroidal hemorrhage, High myopia, Rhegmatogenous retinal detachment, Recombinant tissue plasminogen activator

## Abstract

**Purpose:**

This study aimed to compare the clinical presentation, treatment, and prognosis of two patients with high myopia who suffered rhegmatogenous retinal detachment (RRD) associated with spontaneous suprachoroidal hemorrhage (SSCH) with similar cases discussed in the literature.

**Methods:**

The clinical data of two high myopia patients with RRD associated with SSCH were extracted. A systematic review of the literature was conducted using various databases. The clinical characteristics, treatments, and prognoses of the two cases treated at our hospital were compared with the case reports described in the literature.

**Results:**

Both patients presented at our hospital with elongated axial eye lengths and hypotony. The patients underwent 23G recombinant tissue plasminogen activator (r-tPA)-assisted vitrectomy combined with silicone oil (SO) injection. The literature review identified seven similar cases. The intraocular pressure (IOP) in the literature cases ranged from 2 to 10 mmHg. Seven patients discussed in the literature had elongated axial lengths, of whom six had axial eye lengths measuring over 30 mm. All cases were managed with vitrectomy, and five out of the seven cases subsequently developed proliferative vitreoretinopathy (PVR).

**Conclusion:**

RRD associated with SCH is an exceptionally rare condition, often leading to poor final visual outcomes. Long axial length appears to be the primary risk factor. Most patients present with hypotony, reflecting the underlying pathogenesis. r-tPA–assisted vitrectomy performed within 7 days following diagnosis may help reduce SCH and improve the likelihood of successful retinal reattachment.

## Introduction

Suprachoroidal hemorrhage (SCH) is characterized by bleeding from the posterior ciliary vessels into the suprachoroidal space, which lies between the choroid and the sclera. SCH is a rare, potentially sight-threatening condition that can occur spontaneously or as a complication of ocular surgeries such as cataract extraction, glaucoma surgery, or vitrectomy [1]. Most reported cases of spontaneous suprachoroidal hemorrhage (SSCH) involve patients with hereditary hemodystrophy, age-related macular degeneration, or systemic antithrombotic therapy [2, 3]. In this case study, we report on two patients with high myopia who were treated at our institution for RRD associated with SSCH. Additionally, we conducted a systematic literature review to identify similar cases. The clinical presentation, treatment approaches, and prognosis of the 2 cases treated at our hospital were compared with those reported in the literature to identify risk factors and the optimal management of this rare condition.

## Methods

### Case reporting

The clinical history, treatment, and outcomes of the 2 cases of RRD treated at our institution were extracted from the medical records. Ethical approval was obtained from the Fourth Affiliated Hospital of Zhejiang University School of Medicine and the First Affiliated Hospital of Zhejiang University School of Medicine to extract the data for this case study.

### Search strategy

A systematic literature search was conducted on MEDLINE, PubMed, and Web of Science according to the PRISMA guidelines [4]. The literature was searched using the keywords: [(rhegmatogenous retinal detachment OR retinal detachment) AND (suprachoroidal space OR choroid) AND (hemorrhage) AND (spontaneous)]. Papers published in any language between January 1, 2001, to September 1, 2024, were included. Following the initial screening of retrieved papers, duplicates were removed. Subsequently, full-text articles were independently assessed for eligibility and selected by two reviewers (Yu LB and Ren WN). In cases of disagreement between the two reviewers, a third reviewer (Yan S) was consulted to resolve the discrepancies.

### Inclusion and exclusion criteria

All full-text papers, published in English, that evaluated the presentation of RRD associated with SSCH were included in this review. Studies that did not provide clinical and laboratory features or reported on SCH related to ocular surgery, trauma, and the use of topical medications or anticoagulants were excluded.

### Data extraction

The clinical presentation, treatment provided, and outcomes were extracted from these studies by the reviewer (Yu LB) and reviewed by (Ren WN).

## Results

### Case presentation 1

**Fig. 1:**
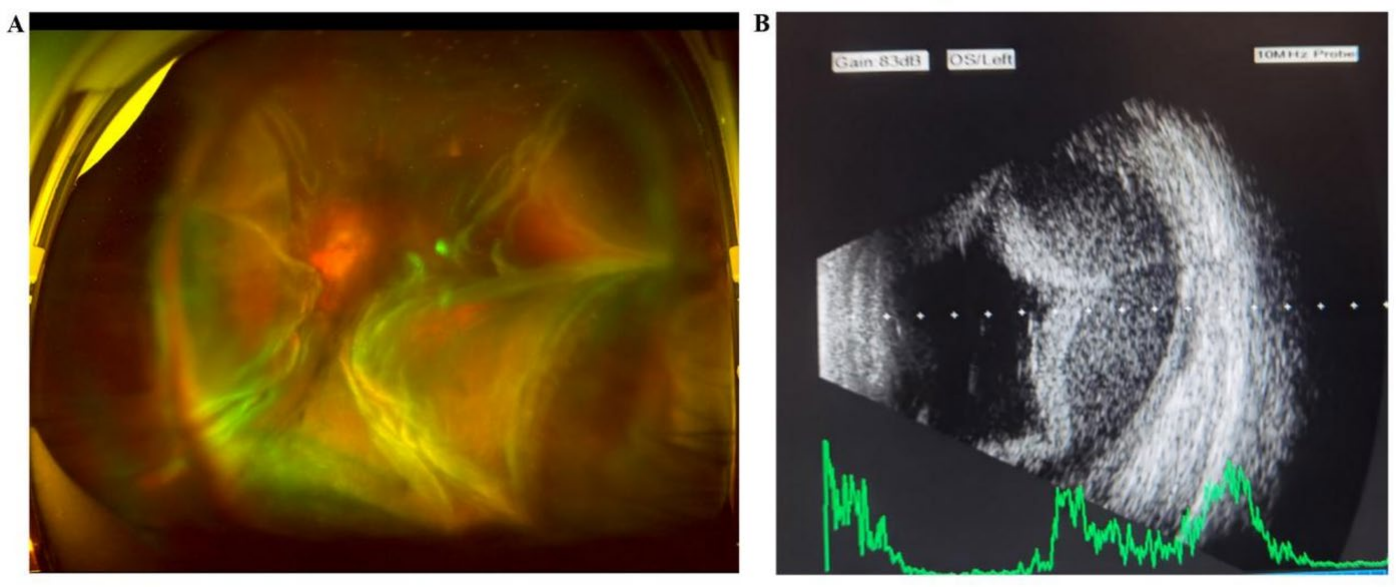
Pre-op images of the left eye for case 1. **A** Pre-op wide-field fundus photograph showing whole retinal detachment with choroidal detachment. **B** B-ultrasound showing retinal detachment with hemorrhagic choroidal detachment

The 53-year-old female presented at the ophthalmology department of the Fourth Affiliated Hospital of Zhejiang University School of Medicine with a one-day history of painful vision loss in her left eye. Her medical history included high myopia for over 40 years and cataract surgery in both eyes performed seven years before this incident. Upon presentation, the patient had a light perception (LP) visual acuity and a low IOP (5 mmHg) in the left eye. Ocular examination revealed conjunctival congestion, slight corneal endothelial folds, and cells in the anterior chamber. Fundus examination showed total retinal detachment with concurrent choroidal detachment in the left eye. B-ultrasound confirmed retinal detachment with significant hemorrhagic choroidal detachment (Fig. 1). Optical coherence tomography (OCT) of the right eye showed normal findings except for the presence of a macular hole. The patient had no history of hypertension, autoimmune diseases, or malignancies, and all laboratory tests were normal. The patient was initially treated with a peri-ball injection of triamcinolone acetonide (20 mg) and intravenous methylprednisolone sodium succinate (500 mg daily for three days). However, after three days, no significant visual improvement was observed.

**Fig. 2:**
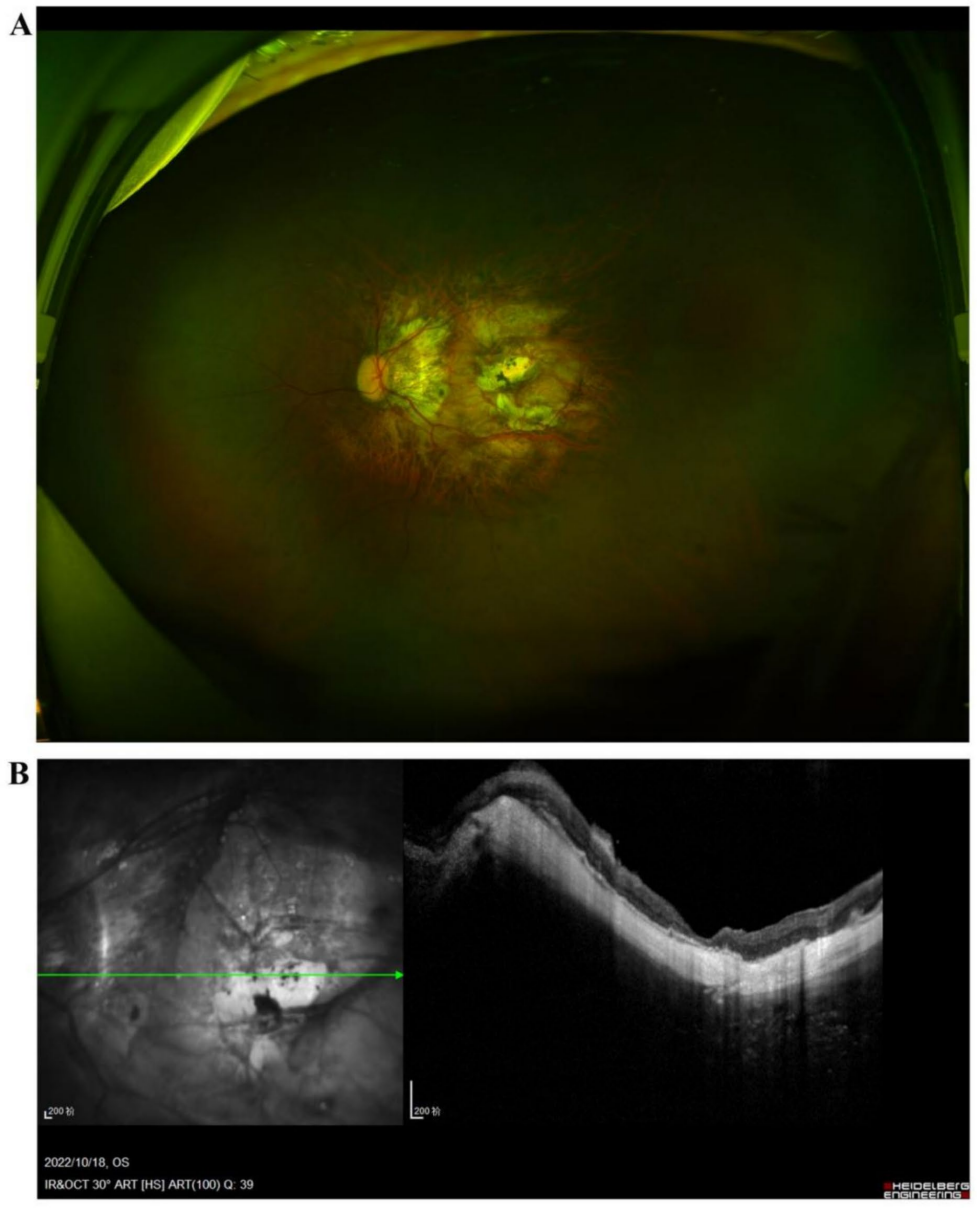
Images of the left eye of case 1 were acquired 1 week after the first surgery. **A** Wide-field fundus photograph showing a reduction in the retinal detachment and choroidal detachment. **B** OCT showing healing of the macular hole

**Fig. 3:**
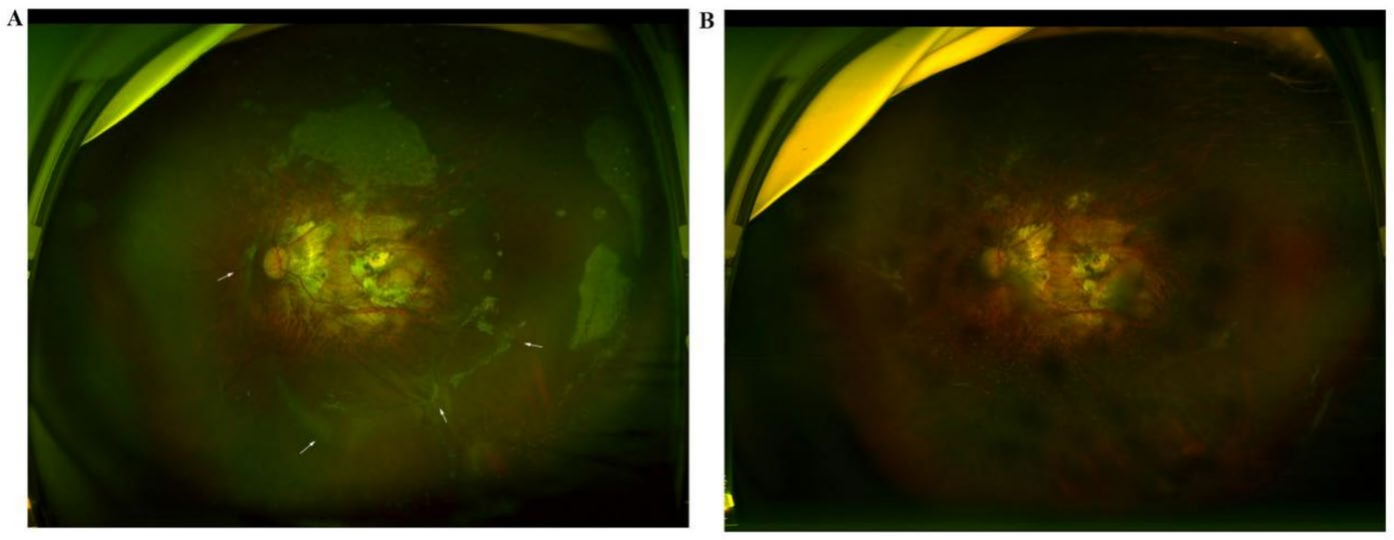
Wide-field fundus photographs of the left eye of case 1. **A** Images acquired 6 weeks after the first surgery showing obvious proliferative vitreoretinopathy in the inferior and nasal retina (white arrows), and **B** Images acquired 5 months after secondary surgery showing the removal of the proliferative membrane and the successful reattachment of the choroid and retina

Five days after the symptom onset, the patient was transferred to the First Affiliated Hospital of Zhejiang University Medical School for surgery. The surgical procedure included recombinant tissue plasminogen activator (r-tPA) suprachoroidal injection, choroidal drainage, 23-gauge vitrectomy, photocoagulation, and silicone oil tamponade. During the surgery, a large volume of the subchoroidal hemorrhage was drained through the scleral incisions. In addition, during the surgical procedure, central macular holes and a peripheral retinal microholes located in the superotemporal retina were identified. A complete vitrectomy with internal limiting membrane peeling using the inverted flap technique was performed to close these holes and improve vision recovery. In addition, a silicone oil tamponade was inserted to support the retinal reattachment. Post-operative examination showed that the retina and choroid were successfully reattached, and the macular hole was closed (Fig. 2). Unfortunately, six weeks later, the patient developed a PVR, which led to recurrent retinal detachment, requiring secondary surgery to remove the proliferative membrane. The surgery resulted in the successful reattachment of the choroid and retina, and the silicone oil was successfully removed. The patient experienced an improvement in the visual acuity from LP to 20/400 (Fig. 3).

### Case presentation 2

**Fig. 4:**
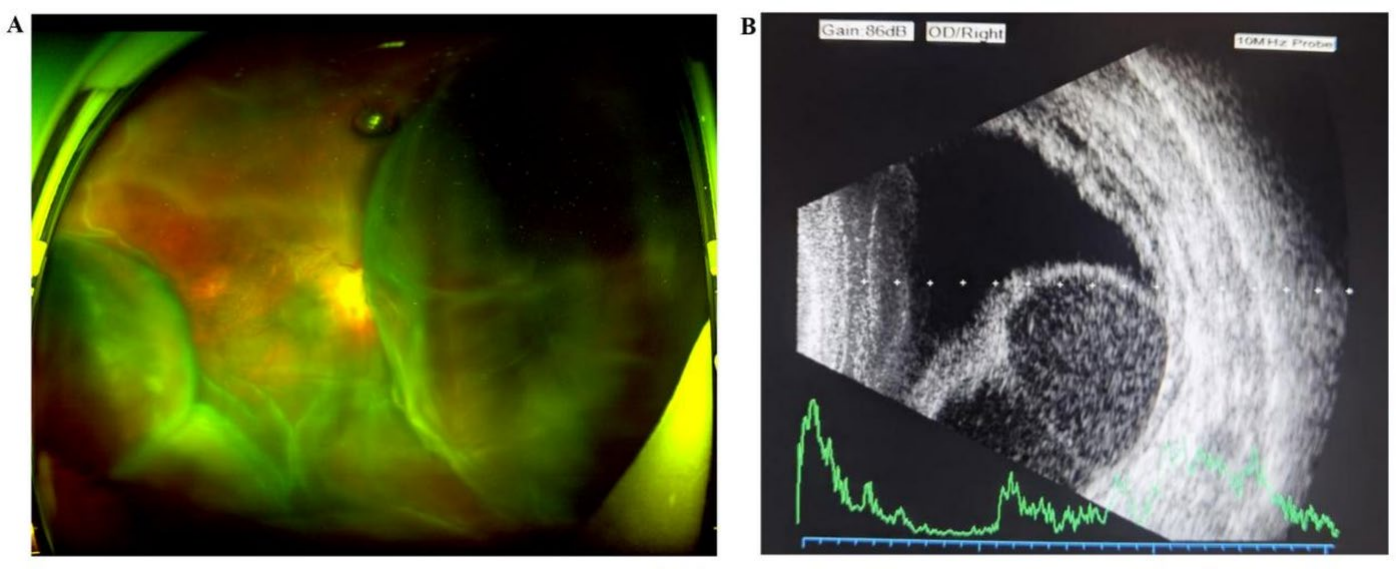
Pre-surgical images of the right eye of case 2. **A** Wide-field fundus photograph showing retinal detachment with choroidal detachment. **B** B-ultrasound showing retinal detachment with hemorrhagic choroidal detachment

**Fig. 5:**
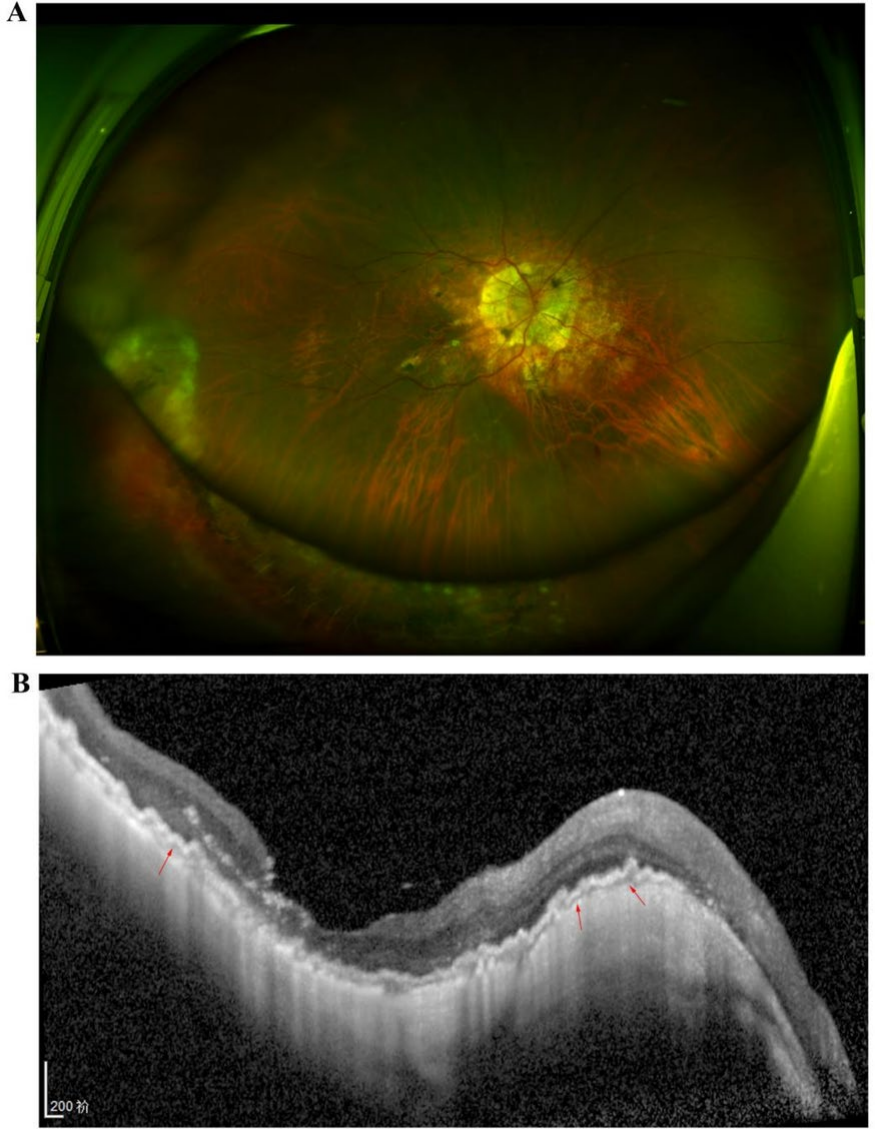
Images of the right eye of case 2 acquired 1 week after the first surgical treatment. **A** Wide-field fundus photograph showing successful reattachment of the retinal and choroidal detachments. **B** OCT showing a reduction in the retinal detachment with some choroidal folds (red arrows)

**Fig. 6:**
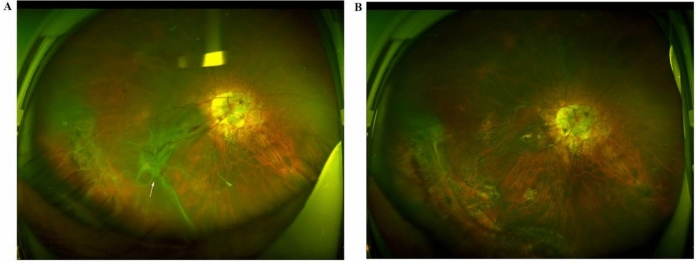
Wide-field fundus photographs of the right eye of case 2. **A** Images acquired 4 weeks after the first surgery showing obvious proliferative vitreoretinopathy in the inferior retina (white arrows). **B** Images acquired 4 months after secondary surgery showing the removal of the proliferative membrane and the successful retinal reattachment

The 68-year-old male presented at the ophthalmology department of the Fourth Affiliated Hospital of Zhejiang University College of Medicine with a two-day history of distorted and painful vision loss in his right eye. His medical history included hypertension, high myopia in both eyes, and cataract surgery in the right eye. The right eye had a low visual acuity (hand motion (HM)) and a low IOP of 3.3 mmHg. Wide-field fundus photography and B-ultrasound indicated RRD combined with SSCH (Fig. 4). The left eye showed no abnormalities apart from a 3 + light brown cataract. The patient was given atropine eye ointment for pupil dilation and received a periocular injection of dexamethasone. The patient was transferred to the First Affiliated Hospital for vitrectomy the following day. The surgical procedure included r-tPA suprachoroidal injection and 23-gauge vitrectomy, choroidal drainage, photocoagulation, and silicone oil tamponade. During the operation, a significant volume of choroidal hemorrhage was drained, and the pre-macular retina was peeled. Intraoperatively, three retinal tears were detected. Specifically, two were small round tears situated in the peripheral temporal retinal region, and the third was found within an inferotemporal degenerative lesion. The retinal breaks were effectively treated with photocoagulation and tamponaded with silicone oil (Fig. 5). At the end of the surgery, a periocular injection of 20 mg triamcinolone was administered. Unfortunately, the PVR led to recurrent retinal detachment within four weeks, necessitating a secondary surgery with membrane peeling and silicone oil reinjection. The silicone oil was successfully removed four months later, with the retina and choroid remaining attached. An improvement in the visual acuity to 20/1000 was also noted (Fig. 6).

### Literature review

**Fig. 7:**
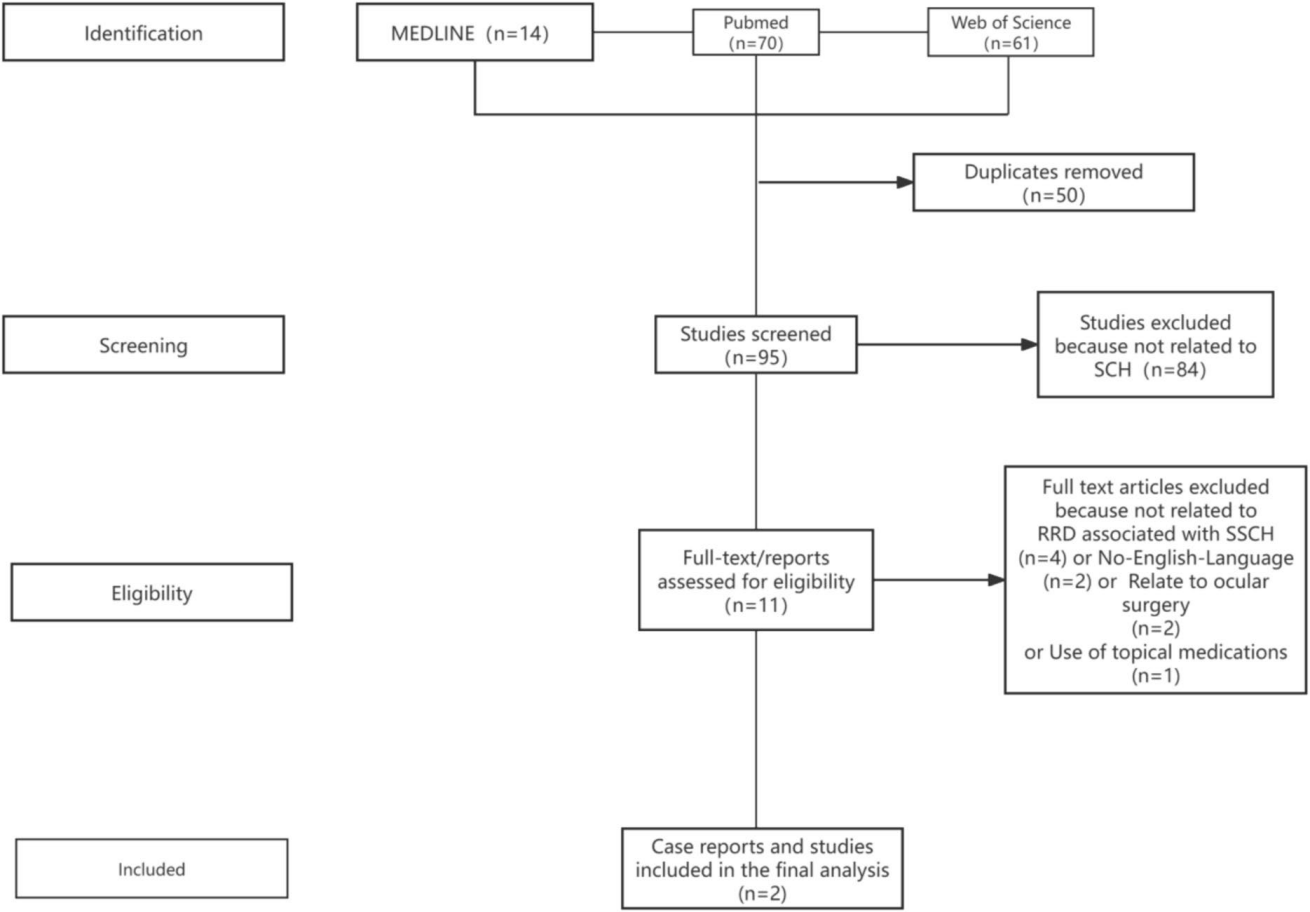
Review process of medical literature related to RRD associated with SSCH

The literature search revealed two studies reporting on RRD associated with SSCH. One study reported [5] on a case of RRD associated with SSCH. The second paper reported on a retrospective study [6] evaluating the clinical courses, risk factors, management strategies, and outcomes of 6 patients who developed this condition following surgery for RRD (Fig. 7). Due to the limited number of cases, the results from these studies were summarized using descriptive statistics.

A total of seven cases of RRD associated with SSCH were identified in the literature. The clinical characteristics of these cases are summarized in Table 1. The ages of the patients ranged from 34 to 73 years, with one patient having a history of hypertension. None of the patients had a history of receiving anticoagulant medications. RRD and suprachoroidal hemorrhage were diagnosed through indirect ophthalmoscopy and B-ultrasound. At the initial presentation, the IOP ranged from 2 to 10 mmHg, and six out of the seven cases presented with hypotony (IOP of ≤ 5 mmHg). The patients’ initial visual acuity ranged from weak light perception (LP) to counting fingers (CF). The mean time from presentation in hospital with visual decline to surgical intervention was 18.14 days (range: 9 to 50 days). Seven patients had elongated axial lengths, and six of these patients had axial eye lengths measuring over 30 mm.

**Table 1:** Clinical characteristics of the cases reported in the literature and the present study

Reference (year)	Age (year)	Sex	Eye	Initial VA	Initial IOP (mmHg)	Final VA
Zhang et al*.* [6]	61	F	R	HM	5	20/100
	53	M	R	CF	2	20/2000
	59	F	R	Faint LP	3	CF
	57	M	L	HM	10	CF
	34	F	R	HM	2	20/200
	59	M	R	CF	4	CF
Chai et al*.* [5]	73	F	L	LP	5	20/2000
*Present study*						
Case 1	53	F	L	LP	5	20/400
Case 2	68	M	R	HM	3.3	20/1000

Reference (year)	AL (mm)	Complications	Intervention duration (days)	SIR	MH
Zhang et al*.* [6]	30.31	None	9	Y	None
	30.39	PVR and TRD	14	N	None
	30.73	PVR and TRD	13	N	HTN
	32.32	PVR and TRD	14	N	None
	28.14	PVR and TRD	15	N	None
	30.03	SG	12	Y	None
Chai et al*.* [5]	> 30	PVR and TRD	50	Y	None
*Present study*					
Case 1	31.91	PVR and TRD	5	Y	None
Case 2	27.25	PVR and TRD	3	Y	HTN

F: Female; M: Male; R: Right eye; L: Left eye; VA: Visual acuity; HM: Hand motion; CF: Counting fingers; LP: Light perception; IOP: Intraocular pressure; AL: Axial length; PVR: Proliferative vitreoretinopathy; TRD: Tractional retinal detachment; SG: Secondary glaucoma; SIR: Silicon oil remove; Y: Yes; N: No; MH: medical history; HTN: hypertension

Most patients underwent a 20G (n = 6) vitrectomy, and one patient underwent a 23G vitrectomy. The suprachoroidal hemorrhage was successfully removed in all 7 patients. Multiple retinal breaks were identified in 6 eyes during the surgery. These breaks were generally located at the periphery of chorioretinal atrophy regions, along the superior and/or inferior temporal retinal vascular arch (n = 4) or the middle of the retinal rim (n = 2). Additionally, one patient developed a 1/5PD hole within the vascular arch below the macula.

Post-operative best-corrected visual acuity (BCVA) improvements were seen in 6 out of the 7 operated eyes and ranged from CF to 20/100. All patients except one developed complications post-surgery. Five patients developed PVR and tractional retinal detachment (TRD), and one patient developed secondary glaucoma (SG). All patients who developed PVR required secondary surgery for membrane peeling and silicone oil tamponade. The silicone oil could not be removed in 4 patients. Furthermore, after surgery, five patients successfully maintained the retinal reattachment.

## Discussion

RRD associated with SSCH is an extremely rare eye disease. Currently, due to the limited clinical data available on this disease, the optimal management remains controversial. In this report, we discuss the clinical characteristics, surgical techniques, and outcomes of two cases of RRD associated with SSCH treated in our hospital and seven other cases discussed in the literature to inform clinical practice.

The precise mechanism of RRD associated with SSCH remains poorly understood. The risk factors for developing SSCH include systemic diseases in individuals receiving anticoagulant therapy [7, 8] and age-related macular degeneration (ARMD) in patients treated with anticoagulants [9, 10]. In our study, only three of the patients were older than 60, and two had hypertension, aligning with these findings. None of the patients had a history of anticoagulant use. Similarly, Chu et al. noted that the association of hypertension, arteriosclerosis, and advanced age with spontaneous hemorrhage has not yet been clarified [11].

RRD with choroidal detachment (CD) makes up approximately 2–5% of all RRD cases [12, 13]. Choroidal detachment in RRD is associated with older age, myopia, prior ocular surgery, multiple retinal breaks, posterior breaks, giant retinal tears, and macular holes. [12, 14–16].

In patients with RRD accompanied by choroidal detachment, multiple retinal breaks are relatively common, with reported rates ranging from 34.6 to 43.8% [14, 15]. Gu et al*.* reported that eyes with RRD and choroidal detachment had a significantly higher incidence of more than three retinal holes (35%) compared with eyes with RRD alone (21%, *P* = 0.044) [14]. In our study, eight patients (88.9%) presented with multiple retinal breaks. These findings suggest that multiple retinal breaks may be more frequent in RRD with SSCH than in RRD with CD. Speaker et al. were the first to report in 1991 that an axial eye length greater than or equal to 25.8 mm was an independent risk factor for the development of SCH during intraocular surgery [17]. In our study, all nine patients had high myopia, with seven patients (77.8%) exhibiting an axial length greater than 30 mm. All patients evaluated in this study were Chinese. Wang et al. reported a high prevalence of myopia in East and Southeast Asia [18]. Gu et al. noted that 34.62% of RRD with CD patients had an axial length of 26.0 mm or higher [14], while Yu et al. [15] reported that 59.70% of RRD with CD had an axial length exceeding 24 mm. Compared with RRD patients with CD, RRD patients with SSCH appear to have a higher prevalence of elongated axial length. This trend is particularly notable when the axial length exceeds 30 mm.

Several studies reported that retinal detachment, ciliary edema, and inflammatory responses can reduce the aqueous secretion, leading to hypotony. Hypotony may exacerbate choroidal detachment by promoting fluid exudation into the tissue space [12, 19]. Yu et al. [15] reported that 36.82% of RRD with CD had an IOP of less than 7 mmHg. Similarly, in our study, all patients except for one presented with hypotony (IOP ≤ 5 mmHg). Compared with RRD with CD, hypotony seems to have a greater impact on RRD patients with SSCH. In patients with high myopia, the elongated axial length subjects the retinal and choroidal tissues to traction and degenerative changes, reducing the ability of choroidal vessels to adapt to intraocular pressure fluctuations [6, 20]. When retinal detachment occurs, the resulting sharp drop in intraocular pressure further increases the risk of SSCH in RRD cases. As a result, in cases of RRD with SSCH, hypotony might be a factor contributing to the pathogenesis of SCH rather than a risk factor.

Several studies evaluated the feasibility of surgically draining the SCH in patients who have not developed RRD. However, to date, there is still no consensus on the optimal timing and benefits of performing this procedure [21, 22]. Some studies recommend waiting 10 to 14 days for the hemorrhagic clot to liquefy [23], while others advocate for early surgical intervention to reduce the risk of TRD and vision loss from chronic atrophy or phthisis bulbi [24]. Zhao et al. [25] described an SCH case treated with tPA-assisted vitrectomy performed five days after the onset of acute massive SCH following cataract surgery. This case achieved favorable visual outcomes. Both patients treated at our hospital underwent r-tPA-assisted vitrectomy within 3–5 days following admission and achieved good anatomical outcomes and an improvement in vision acuity. The interval between admission and surgery ranged between 9 and 50 days in the seven cases described in the literature. Evidence suggests that prolonged duration of appositional SCH correlates with poorer visual outcomes [26]. Therefore, we believe that early diagnosis and r-tPA treatment of RRD associated with SSCH are crucial. We recommend performing surgical drainage within 7 days following the onset of the SCH.

Although surgical intervention for SCH has been proposed as a strategy to enhance treatment outcomes, no randomized trial data or high-quality evidence currently exists to provide clear guidance for its surgical management [27]. The surgical management approaches for SCH range from standalone external drainage to the combination of drainage and PPV [24, 28]. Qureshi et al*.* [29] reported that the mean BCVA improvement was greater in the drainage-only group than in the combined PPV with drainage group. However, the difference was not statistically significant.

Yang et al. [21] suggested that although surgical intervention may relieve pain and elevate the IOP in SSCH patients, it may not necessarily result in improved visual outcomes. However, out of the nine patients discussed in this report, eight achieved improved visual acuity, and three patients achieved 20/400 or better. Several factors lead to limited visual improvement post-surgery, including underlying RRD and high myopia, delayed clot liquefaction, and the high prevalence of PVR. Seven patients in this study developed PVR with TRD, necessitating secondary surgeries such as pre-retinal membrane peeling and temporary or permanent intravitreal silicon oil injection. Temporary silicon oil provides the retinal tamponade necessary for reattachment, facilitates fluid resorption while controlling intraocular inflammation, and can therefore reduce the risk of recurrent SCH [30]. However, it necessitates a second surgery for removal and still provides an incomplete tamponade of the inferior retinal breaks [31]. On the other hand, permanent silicon oil can reduce the risk of retinal detachment in severe PVR, recurrent retinal detachments, or cases where the global integrity of the eye is at risk [32]. Despite its advantages, permanent silicon increases the risk of chronic IOP elevation, silicon emulsification, band keratopathy, corneal decompensation, and long-term optical distortion, potentially limiting visual recovery [33, 34]. Four out of the seven cases discussed in the literature required permanent silicone oil to maintain the attachment of the retina. The two cases treated with early r-tPA-assisted vitrectomy did not require permanent silicone oil. These findings suggest that this procedure may be effective in achieving retinal reattachment without the need for long-term tamponade.

The anatomical success rate of RRD in highly myopic eyes is generally favorable. Kwok et al. reported a primary anatomical success rate of 86.1% in the scleral buckle (SB) group and 75% in the PPV group, with final anatomical success rates ranging from 97.2 and 100%, respectively [35]. PVR is the most common cause of failure following CD repair for RRD [36, 37]. The retinal reattachment rate following conventional scleral buckling remains low due to the high incidence of PVR, varying from 35.4 to 52.4% [12, 38]. However, the introduction of vitrectomy has improved the successful surgical reattachment to 90.5% [39] and reduced the incidence of PVR from about 6.6–28.5% [40–42]. In contrast, in our study, patients with RRD associated with SSCH remain highly prone to develop PVR, with an incidence of 77.8% (7/9). Various factors could potentially increase the risk of PVR in these patients. SCH contains angiogenic factors that contribute to PVR development even after drainage attempts. In addition, blood clots can cause further damage to the retina and exacerbate the inflammatory responses. While r-tPA can accelerate the resolution of blood clots, it does not ensure timely surgical intervention.

Our study has several limitations that have to be acknowledged. Due to the limited number of cases, we could not evaluate the impact of risk factors such as sex, age, systemic therapy, hypertension, and diabetes on the severity of RRD associated with SSCH. The non-randomized design of this study limits the ability to draw definitive conclusions regarding the efficacy of r-tPA-assisted vitrectomy. Therefore, larger multicentre studies are warranted to validate the risk factors for RRD associated with SSCH and to further evaluate the effectiveness of this treatment approach.

## Conclusion

RRD associated with SSCH is an exceptionally rare condition, typically resulting in poor final visual acuity. Long axial length emerges as a primary risk factor. For most patients presenting with hypotony related to disease pathogenesis, r-tPA-assisted vitrectomy performed within 7 days following diagnosis may reduce the SCH and enhance retinal reattachment.

## Data Availability

The datasets used in the current study are available from the corresponding author upon reasonable request.
